# Study of the sorting pattern of dredger fill under artificial disturbance based on the concealment degree

**DOI:** 10.1038/s41598-022-11416-0

**Published:** 2022-05-06

**Authors:** Yuan Yanzhao, Zhai Juyun

**Affiliations:** College of Civil and Traffic Engineering, Henan University of Urban Construction, Pingdingshan, 467036 China

**Keywords:** Environmental sciences, Solid Earth sciences

## Abstract

This paper focuses on the sorting pattern of silt deposited at estuaries under artificial disturbance based on the concept of bidirectional concealment, which is introduced for such silt with similar particle size, low disturbance exposure angle, and difficult sorting. By establishing the relationship among the absolute concealment (Δ_i_), the drag force coefficient (${C}_{\mathrm{D}}$), and the lift force coefficient (${C}_{\mathrm{L}}$) of highly concealed silt particles under disturbance and considering the concepts of disturbance intensity, disturbance direction, and particle concealment degree, the contribution equation for the effective depth (Y) of artificial disturbance in the deposited slurry is introduced, and the mechanical equation for the internal disturbance of silt is established. On this basis, the motion initiation probability for fine particles (${\varepsilon }_{{d}_{i}}$) is calculated using the Shields parameter ($\Theta$c), and the theoretical model of plastic sandy silt sorting under artificial disturbance is derived from the Markov chain-based three-state transition. The method proposed in this paper can explain the bedload gradation of sandy silt under internal physical disturbance. The calculated data in this paper agrees well with that of the flume test on uniform sandy silt and the related sediment particle theories. Therefore, the clay particle transport pattern model in slurry under artificial disturbance based on the bidirectional concealment degree can explain the gradation changes of flow-plastic sandy silt under artificial disturbance, thus providing a theoretical basis for the research on the sorting of flow-plastic sandy silt under artificial disturbance.

## Introduction

With the development of its national economy, land reclamations in China’s coastal cities have increased dramatically. However, the commonly used land reclamation technologies are applied on the sandy silt deposited on the seabed at the estuaries. As fine-grained soil, sandy silt has low loading efficiency for the transport ships and long consolidation periods after hydraulic filling, which poses significant challenges for the subsequent construction projects. Research on the deposition process of this unique dredger fill material by Jia et al.^1^. revealed that applying artificial disturbance to the deposited flow-plastic sandy silt during dredger filling can have many advantages, such as removing the clay particles from the slurry, promoting sediment armoring, and effectively improving the early stage strength and density of the sandy silt.

Research on bedload transport of clay-sand mixtures under disturbance can be generally divided into two types. The first type adopts the calculation model based on the principle of sediment balance in slurry, which is calculated according to the maximum particle size for motion initiation, sediment transport rate, and particle transport gradation. Gessler^2^ adopted the method of probability theory to analyze the movement probability of different gradations during riverbed armoring and derived the calculation model of natural riverbed load gradation. Based on the same model, He et al.^3^ analyzed the position characteristics of ununiform sand and obtained the equation to calculate the maximum particle size for bedload motion initiation with *medium transport* as the motion initiation threshold. Nie et al.^4^ compared the conceptual flume test results of riverbed scouring with the sediment transport rates calculated using the equations of Meyer^5^ and Bagnold^6^, concluding that the sediment transport rate of bedload was positively correlated with the peak disturbing force. After studying irregular sand grains, Dey and Ali^7^ obtained the tangential stress equation under three motions, i.e., rolling, sliding, and saltation, and introduced the mechanistic-cum-stochastic theory to analyze the entrainment probability under these three modes. The second type is based on the slurry disturbance layer theory or active layer theory. First, the size of the disturbance area generated during the disturbance process is determined, and the thickness of the disturbance layer is calculated based on that of the disturbance area. Then, the disturbance and scouring changes in the slurry can be analyzed usually by introducing the concealment coefficient of ununiform sand to correct the non-motion initiation probability of clay and sand. With the modified Shields curve, model analysis of the transport probability pattern of Gessler particles can be conducted^2^. Thus, the transport pattern of clay and sand particles and the particle size of bedload motion initiation under disturbance can be obtained.

Therefore, the transport pattern of bedload such as clay and sand can be theoretically analyzed based on the particle mechanical balance theory and the probability and statistics theory. However, compared with natural river scouring, the sorting of deposited dredger fill under artificial disturbance is unique since the disturbance is artificial and occurs in the weak stratum after silt deposition. Therefore, previous studies based on the moment balance of sand particles and the effects of particle exposure and concealment degrees in the disturbance layer, even those analyzing the transport of bedload and the resanding pattern with the probability theory, could not explain the transport pattern of fine particles in plastic sandy silt under the outward turbulence generated by artificial disturbance. The reason is the lack of resanding found at the bottom of natural riverbeds and the multidirectional nature of the scouring force. Therefore, the concept of the bidirectional concealment degree of sediment scouring (${\Delta }_{x}^{\mathrm{^{\prime}}},{\Delta }_{z}^{\mathrm{^{\prime}}}$) is proposed. Then, force analysis is conducted on the clay and sand particles under the disturbance force considering the bidirectional concealment degree. The probability of clay particle suspension and sand particle deposition is analyzed, and a transport pattern model of clay particles in slurry under artificial disturbance is finally established. Test results of Jia et al.^1^ were collected to comparatively analyze the armoring gradation and bedload gradation of sandy silt slurry after disturbance and the particle transport pattern under different disturbance conditions. The proposed model can provide certain theoretical support for the treatment technology of dredger fill foundations.

## Analysis of the concealment degree of sandy silt

The materials studied in this paper are soil particles carried to and widely deposited at estuaries. Due to water scouring, the soil particles have poor particle size distribution around 0.1 mm. Specifically, the proportion of sand particles between 0.1 and 0.5 mm is over 50%, and the content of silt particles between 0.074 and 0.1 mm is over 30%. As the clay particles (d_i_) and sand particles (d_m_) in sandy silt are similar in size and d_i_ ≤ d_m_, the sand particles on the surface of the disturbance layer in the thick slurry are concealed by the clay particles. In this case, the clay particles are more susceptible to motion initiation by the disturbing force. Therefore, the absolute concealment degree (Δ_i_) between particles is defined as the vertical distance of the sand particles on the surface of the disturbance layer to the highest point in the direction of the disturbance. Since the disturbance surface is curved and clay and sand particles are randomly arranged on it, the traditional vertical exposure degree of clay and sand particles under horizontal flow cannot fully reflect the relationship between clay and sand particles under artificial disturbance. That is, there are both vertical exposure and horizontal exposure on the cross-section of disturbance^8^.

### Vertical relative exposure ${{\varvec{\Delta}}}_{{\varvec{z}}}^{\mathbf{^{\prime}}}$

The different shapes of soil particles can lead to frictions between them and different trends of their transport. However, the study of particle concealment mainly focuses on the effects of scouring on the particles^8^. Simplifying the particle friction by assuming the soil particles as spheres can effectively quantify the particle concealment degree. According to previous research^9–11^, when a slurry of uniform particles is flowing upward, the vertical relative exposure between the particles (${\Delta }_{z}^{^{\prime}}$) is the ratio of the mutual concealment distance between two adjacent particles in the direction of water flow to the radius of the particle, i.e.,${{ \Delta }}_{z}^{^{\prime}} = 2\left| {AB} \right|$/D. The probability distribution equation is as follows:

1 $$F\left( {\Delta_{z}^{^{\prime}} } \right) = \left\{ {\begin{array}{*{20}l} {1,} \hfill & {\left( {\Delta_{z}^{^{\prime}} > 1} \right),} \hfill \\ {\frac{{\Delta_{z}^{^{\prime}} - 0.134}}{0.866},} \hfill & {\left( {\Delta_{zw}^{^{\prime}}\leqslant \Delta_{z}^{^{\prime}}\leqslant 1} \right),} \hfill \\ {0,} \hfill & {\left( {\Delta_{z}^{^{\prime}} < \Delta_{zv}^{^{\prime}} } \right),} \hfill \\ \end{array} } \right.$$

**Figure 1:**
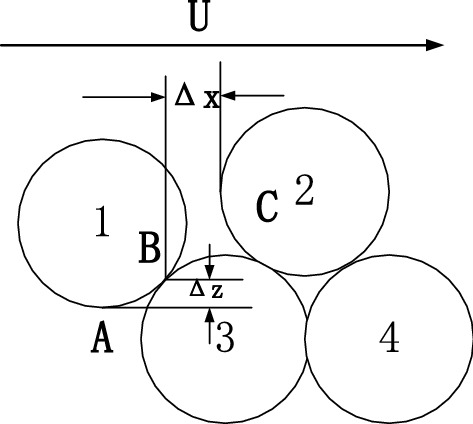
The schematic diagram of exposure.

When ${\Delta }_{z}^{^{\prime}}$ > 1, the probability distribution function of vertical relative exposure is 1, and the particles are at the compressed state, which is an inevitable event. When ${\Delta }_{z}^{^{\prime}}$ = 1 ($\Delta_{z} D/2$), the clay and sand particles are completely concealed, as shown in Fig. 1, where particle 4 is completely concealed by particle 3. When ${{ \Delta }}_{z}^{^{\prime}} < {\Delta }_{zv}^{^{\prime}}$, the probability distribution function of vertical relative exposure is 0, and the slurry particles are at the dispersed state. As demonstrated by particles 1 and 3, the relative exposure between them is 0. When $\Delta_{z}^{^{\prime}} \Delta_{zm}^{^{\prime}}$ (${\Delta }_{zm}^{^{\prime}} = 0.134$), particles are closely arranged and at the lower limit state, as shown in Fig. 1.

The expected vertical relative exposure is:

2 $$\overline{{\Delta_{z}^{^{\prime}} }} = E\left( {\Delta_{z}^{^{\prime}} } \right) = \mathop \smallint \limits_{{\Delta_{i = n}^{^{\prime}} }}^{1} \Delta_{z}^{^{\prime}} {\text{d}}F\left( {\Delta_{z}^{^{\prime}} } \right) = \frac{{1 - \left( {\Delta_{zm}^{^{\prime}} } \right)^{2} }}{{2\left( {1 - \Delta_{zm}^{^{\prime}} } \right)}} = \frac{{1 + \Delta_{zm}^{^{\prime}} }}{2} = 0.567$$

### Horizontal relative exposure

The disturbance in sandy silt flowing through the particles creates swirling structures between them in the process, which not only exerts a vertical force on the particles but also causes horizontal lift and drag forces between the particles. As a result, the particle arrangement is different, i.e., the ratio of horizontal particle transport distance to particle radius is the horizontal relative exposure ${\Delta }_{x}^{^{\prime}} = 2\left| {BC} \right|/D$. For the disturbance layer in thick slurry,${{ \Delta }}_{x}^{^{\prime}}$ among the particles is the same as ${\Delta }_{z}^{^{\prime}}$. Therefore, all particles are assumed to be closely arranged. In addition, ${\Delta }_{x}^{^{\prime}}$ and ${\Delta }_{z}^{^{\prime}}$ change with the changes of particle positions. According to the analysis of probability distribution, the correlation between ${\Delta }_{x}^{^{\prime}}$ and ${\Delta }_{z}^{^{\prime}}$^12^ can be further defined as:

3 $${\Delta }_{lx}^{^{\prime}} = \left( {\overline{D}/D_{l} } \right)\sqrt {2{\Delta }_{lz}^{^{\prime}} - {\Delta }_{lz}^{{{^{\prime}}2}} }$$

Due to the similar sizes of clay and sand particles in sandy silt, ${\Delta }_{x}^{^{\prime}}$ should fall in the range of $0.5\overline{D}/D_{l}\leqslant \Delta_{lx}^{^{\prime}}\leqslant \overline{D}/D_{l}$. Thus, the probability distribution function of ${\Delta }_{x}^{^{\prime}}$ in sandy silt^8^ can be defined as:

4 $$F\left( {\Delta_{x}^{^{\prime}} } \right) = \left\{ {\begin{array}{*{20}l} {1,} \hfill & { \left( {\Delta_{x}^{^{\prime}} > 1} \right),} \hfill \\ {\frac{{0.866 - \sqrt {1 - \left( {\Delta_{x}^{^{\prime}} } \right)^{2} } }}{0.866},} \hfill & {\left( {0.5\leqslant\Delta_{x}^{^{\prime}} \leqslant1} \right)} \hfill \\ {0,} \hfill & {\left( {\Delta_{x}^{^{\prime}} < 0.5} \right).} \hfill \\ \end{array} } \right.$$

Its horizontal probability distribution indicates that the expected horizontal relative exposure is:

5 $$\overline{{\Delta_{x}^{^{\prime}} }} = E\left( {\Delta_{x}^{^{\prime}} } \right) = \mathop \smallint \limits_{0.5}^{1} \Delta_{x}^{^{\prime}} {\text{d}}F\left( {\Delta_{x}^{^{\prime}} } \right) = 0.855.$$

### Relationship between the relative exposure degree and the drag force and lift force coefficients of water flow

**Figure 2:**
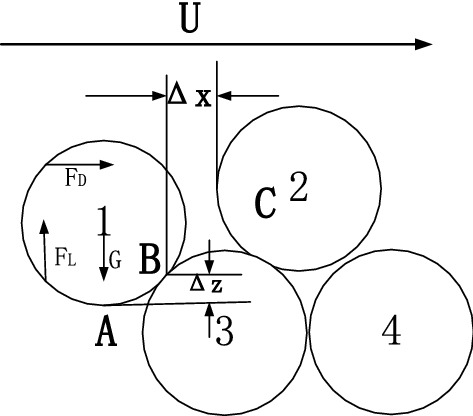
The schematic diagram of the stress on particles.

According to previous research^12,13^, the vertical relative exposure ${\Delta }_{z}^{^{\prime}}$ and horizontal relative exposure ${\Delta }_{x}^{^{\prime}}$ in the concept of bidirectional exposure are as shown in Fig. 2. In addition, ${\Delta }_{x}^{^{\prime}} = \frac{{2{\Delta }_{x} }}{D}$ and ${\Delta }_{z}^{^{\prime}} = \frac{{2{\Delta }_{z} }}{D}$ represent horizontal relative exposure and vertical relative exposure, respectively. Bai et al.^8^ conducted simulation research and obtained the relationship between the drag force and lift force coefficients and the vertical relative exposure of particles, as shown in Fig. 2.

6 $$C_{{\text{D}}} = - 1.306 + 11.617{\Delta }_{x}^{^{\prime}} - 17.511\left( {{\Delta }_{x}^{^{\prime}} } \right)^{2} + 8.417\left( {{\Delta }_{x}^{^{\prime}} } \right)^{3}$$

7 $$C_{{\text{L}}} = - 2.378 + 14.994{\Delta }_{x}^{^{\prime}} - 21.616\left( {{\Delta }_{x}^{^{\prime}} } \right)^{2} + 9.95\left( {{\Delta }_{x}^{^{\prime}} } \right)^{3} + 26.924{\text{exp}}\left( { - 3.534 \times 10^{ - 4} {\text{Re}}_{*} } \right)$$

As ${\Delta }_{x}^{^{\prime}} = \sqrt {2{\Delta }_{z}^{^{\prime}} - \left( {{\Delta }_{z}^{^{\prime}} )} \right.^{2} }$,the correlation between the drag force coefficient $C_{{\text{D}}}$ and the vertical relative exposure ${\Delta }_{z}^{^{\prime}}$ are obtained as follows:

8 $$C_{{\text{D}}} = - 1.306 + 11.617\sqrt {2{\Delta }_{{\text{z}}}^{^{\prime}} - \left( {{\Delta }_{z}^{^{\prime}} } \right)^{2} } - 17.511\left[ {2{\Delta }_{z}^{^{\prime}} - \left( {{\Delta }_{z}^{^{\prime}} } \right)^{2} } \right] + 8.417\left[ {\sqrt {2{\Delta }_{z}^{^{\prime}} - \left( {{\Delta }_{z}^{^{\prime}} } \right)^{2} } } \right]^{3}$$

$C_{{\text{D}}}$ and ${ }C_{{\text{L}}}$ can be calculated by bringing Eqs. (2) and (5) into Eqs. (7) and (8) as 1.11 and 0.86, respectively.

## Particle migration model of sandy silty soil under artificial disturbance

**Figure 3:**
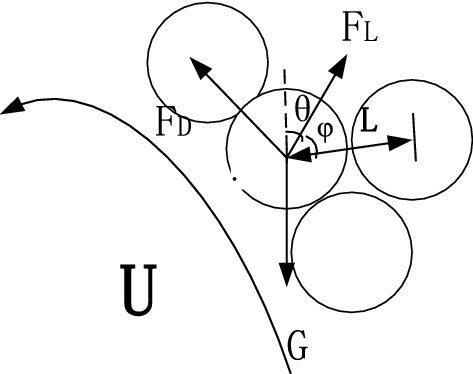
The force analysis of particles in the slurry.

The disturbance conditions and the relative positions of clay and sand particles are prerequisites for determining the motion initiation of clay particles. However, the relative positions of clay and sand particles in sandy silt under disturbance can be analyzed based on the concealment degree, as shown in Fig. 3. The drag force $F_{{\text{D}}}$ and lift force $F_{{\text{L}}}$ can be obtained via theoretical analysis. In addition, the disturbance flow follows the Gaussian distribution^14^. According to the relevant theories and research^11–14^, the exposure angle $\varphi_{0}$, the directional angle of drag force $\theta_{0}$, and the relative gravity arm ${ }L_{1}$ between the particles can be obtained. Thus, the transport of clay particles among sand particles can be analyzed.

### Motion initiation probability of clay particles

**Figure 4:**
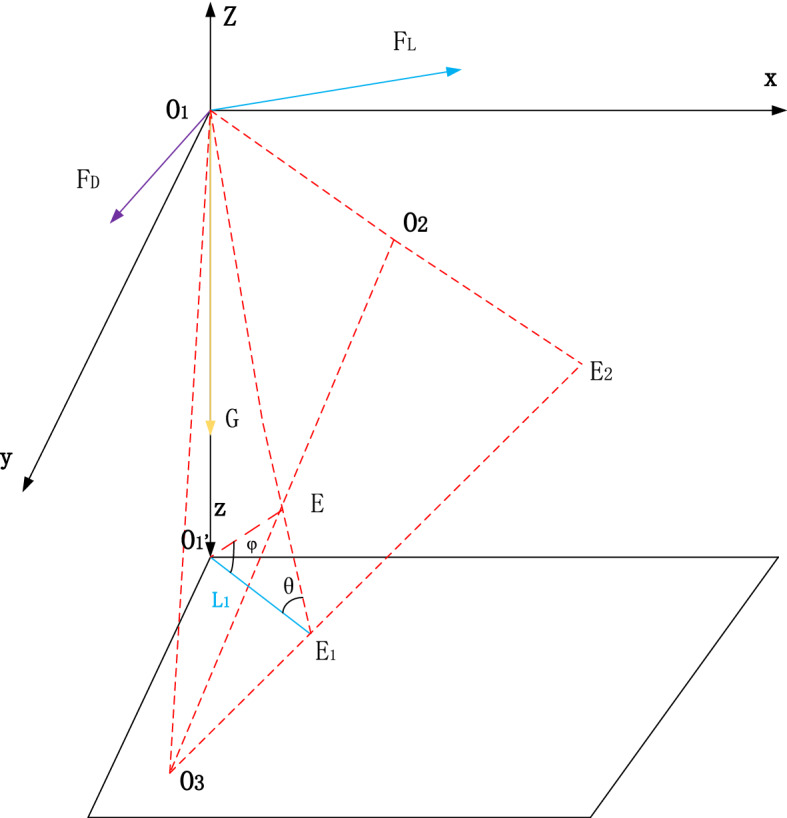
The force analysis diagram of particle O_1_.

As shown in Fig. 3, the clay particles under disturbance are subjected to drag force $F_{{\text{D}}}$, lift force $F_{{\text{L}}}$, and gravitational force $G$^15^, as shown in Fig. 4, which can be expressed as:

9 $$\left\{ {\begin{array}{*{20}c} {F_{{\text{D}}} = \frac{{C_{{\text{D}}} \rho }}{2}\frac{\pi }{4}D^{2} V_{{{\text{b}}.{\text{e}}}}^{2} } \\ {F_{{\text{L}}} = \frac{{C_{{\text{L}}} \rho }}{2}\frac{\pi }{4}D^{2} V_{{{\text{b}},{\text{e}}}}^{2} } \\ {G = \left( {\rho_{{\text{s}}} - \rho } \right)g\frac{\pi }{6}D^{3} } \\ \end{array} } \right.$$

where $C_{{\text{D}}}$ is the drag force coefficient, $C_{{\text{L}}}$ is the lift force coefficient, $V_{b,e}$ is the instantaneous velocity of fluid motion initiation, ${ }\rho_{{\text{s}}}$ is the density of clay particles, $\rho$ is the density of water; and *D* is the particle diameter.

As shown in Fig. 4, clay particle O_1_ on the surface of the disturbance layer is taken as the research object, the sphere centers of subsurface sand particles O_2_ and O_3_ are taken as the virtual fulcrum, and O_1_ and O_2_ are taken as the virtual arm $L_{1}$. The saltating motion initiation equation can be expressed as follows:

10 $$F_{D} {\text{cos}}\varphi_{0} \cdot L_{1} \cdot {\text{tan}}\theta_{0} + F_{{\text{L}}} sin\varphi_{0} \cdot L_{1} cot\theta_{0} = GL_{1}$$

The instantaneous velocity of the initial disturbance calculated with Eq. (10) is the threshold velocity $V_{b,e}$ for particle motion initiation, which can be expressed as:

11 $$V_{b,e} = \sqrt {\frac{{\frac{4}{3}\left( {\rho_{s} - \rho } \right)}}{{C_{{\text{D}}} \left[ {{\text{cos}}\varphi_{0} {\text{tan}}\theta_{0} } \right]\rho + C_{{\text{L}}} \left[ {sin\varphi_{0} cot\theta_{0} } \right]\rho }}gD}$$

where $\rho_{s}$ is the density of sand particles, $\rho$ is the density of clay particles, and $D$ is the particle diameter.

According to previous research^16^, the ratio of the average velocity of the disturbance flow $\overline{{V_{{\text{b}}} }}$ to the friction velocity $V_{v}$ follows logarithmic distribution^10^:

12 $$\frac{{\overline{{V_{{\text{b}}} }} }}{{V_{v} }} = 5.75{\text{log}}\left( {30.2\frac{y\chi }{{k_{{\text{x}}} }}} \right)$$

where $V_{v}$ is the friction velocity, $k_{{\text{x}}}$ is the equivalent roughness; $\chi$ is a coefficient ($\chi = 1{\text{ D}}$ for rough disturbance surface of uniform particles), and y is the distance from the disturbance center.

Assuming that y = 1D, the relationship between the average velocity of the disturbance flow $\overline{{V_{{\text{b}}} }}$ and the Shields parameter ${\Theta }$ can be obtained from the simultaneous equations of Eq. (12) and Shields parameter ${\Theta }$^14^:

13 $$\overline{{V_{{\text{b}}} }} = 8.51\sqrt {\Theta } \sqrt {\frac{{\rho_{{\text{s}}} - \rho }}{\rho }gD}$$

According to Eq. (13), particles with a smaller concealment degree ${\Delta }_{z}^{^{\prime}}$, higher position of surface particles relative to subsurface particles, and lower density are more prone to motion initiation by a constant disturbance force. With a smaller directional angle, the drag force has a greater component in the direction of particle rolling, and the particles are easier to be entrained. Therefore, under constant flow conditions, the motion initiation probability $\varepsilon$ of clay particles in the disturbance layer not considering the random distribution can be expressed as:

14 $$\varepsilon = \mathop \smallint \limits_{ - \Delta i}^{\Delta i} \left[ {1 - \mathop \smallint \limits_{0}^{\Delta i} F\left( {\Delta_{z}^{^{\prime}} } \right){\text{d}}\Delta_{z}^{^{\prime}} } \right]F\left( {\Delta_{x}^{^{\prime}} } \right){\text{d}}\Delta_{x}^{^{\prime}}$$

where $F\left( {{\Delta }_{z}^{^{\prime}} } \right)$ and $F\left( {{\Delta }_{x}^{^{\prime}} } \right)$ can be calculated with Eqs. (2) and (5).

The physical implication of Eq. (14) is as follows. Without considering the randomness of the water flow and the directional angle variation of the drag force, if particles with a vertical exposure degree of ${\Delta }_{z}^{^{\prime}}$ can be entrained in the slurry, then all clay particles with exposure degrees in the range of $\Delta_{zw}^{^{\prime}} \Delta_{z}^{^{\prime}} 1$ can be entrained. Under a constant exposure angle, if clay particles with horizontal concealment degree of ${\Delta }_{x}^{^{\prime}}$ can be entrained, particles with horizontal concealment degrees in the range of $0.5\Delta_{x}^{^{\prime}} 1$ can be entrained.

In addition to the randomness of particle position, the randomness of particle motion initiation is also ascribed to the randomness of instantaneous flow velocity. Thus, the motion initiation probability of surface clay particles $\varepsilon_{s}$ can be expressed as:

15 $$\varepsilon_{s} = \left[ {1 - \mathop \smallint \limits_{0}^{{a_{0} }} f\left( X \right){\text{d}}X} \right] \cdot \mathop \smallint \limits_{0.5}^{1} \left[ {1 - \mathop \smallint \limits_{{\Delta_{zw}^{^{\prime}} }}^{1} F\left( {\Delta_{z}^{^{\prime}} } \right){\text{d}}\Delta_{z}^{^{\prime}} } \right]F\left( {\Delta_{x}^{^{\prime}} } \right){\text{d}}\Delta_{x}^{^{\prime}}$$

where the instantaneous velocity of the bottom water flow follows a normal distribution, which can be expressed as $X = \left( {V_{b,e} - \overline{{V_{{\text{b}}} }} } \right)/0.37\overline{{V_{{\text{b}}} }}$ after standard normalization. When ${\Theta } = 0.3$, the calculated motion initiation probability of clay particles in the disturbance layer $\varepsilon_{s}$ is 0.97, which is approximately equal to the calculated results of Sun and Donahue^17^ and Armanini et al.^18^, but slightly smaller than the results calculated with Einstein’s formula^19^.

Other than the surface, clay particles are also inside the disturbance layer, and their motion initiation probability can be expressed as:

16 $$\varepsilon_{{\text{T}}} = \varepsilon_{s} \varepsilon_{{\text{b}}} + \varepsilon_{{\text{m}}} \varepsilon_{{\text{d}}}$$

where $\varepsilon_{{\text{T}}}$ is the motion initiation probability of clay particles on the disturbance layer, $\varepsilon_{{\text{b}}} = 0.389$, $\varepsilon_{{\text{d}}} = 1 - \varepsilon_{{\text{b}}}$, and ${ }\varepsilon_{{\text{m}}}$ is the motion initiation probability of particles in the disturbance layer. With the motion initiation stress model of a single clay particle adopted in this paper, which is suitable for analyzing the clay particle motion initiation stress when the Shields parameter is below the motion initiation condition of the stratification flow, the water flow causes the motion initiation of surface clay particles but not the sand particles. Hence $\varepsilon_{{\text{m}}} = 0$.

**Figure 5:**
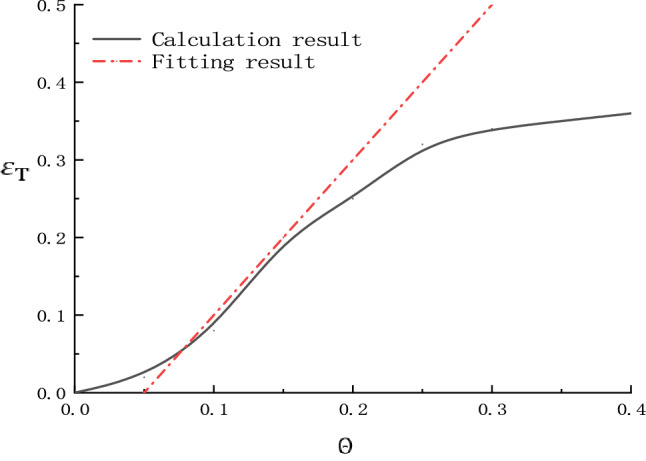
Correspondence between ${\varepsilon }_{\mathrm{T}}$ and $\Theta$.

According to Eq. (16) and relevant research^8,14^, when C_D_ = 0.4, C_L_ = 0.1, $\varepsilon_{{\text{b}}}$ = 0.389, and $\varphi_{0 }$ = 0, the different Shields parameters and the motion initiation probability of clay particles can be calculated, as shown in Fig. 5.

Shields parameter Θ is the ratio of the motion initiation force of particles to river bed resistance. A larger Shields parameter Θ means a greater probability of particle motion initiation, which can be expressed as follows:

17 $${\Theta } = \frac{{\tau_{0} }}{{\left( {\gamma_{{\text{s}}} - \gamma } \right)D}}$$

where $\tau_{0}$ is the shear stress on the bed surface, $\gamma_{{\text{s}}}$ is the bulk density of the sediment, $\gamma$ is the bulk density of water, and $D$ is the average particle size of the sediment.

Analysis of the relationship between clay particles on the disturbance layer and Shields parameter ${\Theta }$ shows that under the above conditions, the critical motion initiation threshold ${\Theta }$ c is above the range of 0.25–0.3. At the motion initiation threshold, the motion initiation probability $\varepsilon_{{\text{T}}}$ is close to 0.389, and the corresponding motion initiation probability on the disturbance surface is 1.

### Determination of the velocity and motion layers of clay particles

The transport of different particles is analyzed according to the findings of the sediment study. In this paper, the Englund formula^19^ is used to study the velocity of clay particles, which can be expressed as:

18 $$V_{b,e} /V_{v} = k_{0} \left( {1 - 0.7\sqrt {{\Theta }_{{\text{c}}} /{\Theta }} } \right)$$

where coefficient $k_{0}$ is set as 9.3 by Englund^19^. According to the previous research^18,20^, ${\Theta }_{{\text{c}}}$ is between 0.03 and 0.06. In this paper, the *medium transport* motion initiation threshold suggested by Dou^16^ is adopted, i.e., $\varepsilon_{{\text{T}}} = 0.0228$, and the corresponding ${\Theta }_{{\text{c}}}$ is 0.035. Thus, Eq. (18) is adopted for the subsequent research.

Despite the extensive research on sediment transport stratification, it can not be accurately determined by the time of writing. Therefore, the findings of Zhen et al. (2016) are adopted. Specifically, when Shields parameter ${\Theta }$ is below the motion initiation threshold, the number of disturbance layers is 1. Otherwise, the number of layers depends on the effective flow condition (${\Theta }$–${\Theta }_{{\text{c}}}$) and the motion initiation threshold of the stratification flow (${\Theta }_{{{\text{cf}}}}$). additionally, it is assumed that:

19 $$N = k_{N} \left( {\frac{{{\Theta } - {\Theta }_{c} }}{{{\Theta }_{cf} }}} \right)$$

When ${\Theta }_{cf} = 0.3$, C_D_ = 0.4, C_L_ = 0.1, P_b_ = 0.389, and $\varphi = 0$, $k_{N}$ can be calculated as 0.75 by introducing Eq. (18) into Eq. (19) and summarizing the experimental data of Meyer-peter^5^ and Murphy^21^ according to Wang et al.^22^.

Gradation after the scouring caused by artificial disturbance can be calculated by probability models. Assuming the total amount of clay and sand particles before disturbance is 1 (absolute value), the remaining amount of clay particles of a certain diameter after several physical disturbances can be calculated with the following equation^23^:

20 $$S_{i} = \left( {1 - \varepsilon_{{d_{i} }} } \right)p_{0i}$$

where $S_{i}$ is the amount of the sediment of certain particle size, $\varepsilon_{{d_{i} }}$ is the motion initiation probability of clay particles with a corresponding particle size of $d_{i}$, and $p_{0i}$ is the weight percentage of sand particles in the *i*th particle size group $d_{i}$ in the initial slurry.

Therefore, by accumulating the number of sand particles in each particle size grade, a probability calculation model of the sand gradations below a certain particle size grade $d_{i}$:

21 $$P_{i} = \frac{{\mathop \sum \nolimits_{i = 1}^{k} \left( {1 - \varepsilon_{{d_{i} }} } \right)p_{0i} }}{{\mathop \sum \nolimits_{i = 1}^{m} \left( {1 - \varepsilon_{{d_{i} }} } \right)p_{0i} }} = \frac{{S_{k} }}{{S_{m} }}$$

where $P_{i}$ is the percentage of particles smaller than $d_{i}$ in the disturbance layer, i = 1 is the minimum particle size $d_{{{\text{min}}}}$, i = k is the clay particle size, and i = m is the sand particle size.

Since the effects of artificial disturbance on the sediment are quite different, the sediment gradation differs significantly. Therefore, the exchange of sediment is mostly through the downward entrenchment of river waves. In contrast, the gradation of silt is relatively narrow, and the artificial disturbance forms an upward and outward entrenchment. As a result, the disturbance layer is relatively low, thus promoting the discharge and exchange of clay particles. Specifically, the lower clay particles under disturbance gradually rise to the upper layer. Thus, the sediment balance equation of^23^ can be transformed as:

22 $$Z^{\left( n \right)} P_{{{\text{ai}}}}^{{\left( {n - 1} \right)}} - H_{{\text{s}}}^{\left( n \right)} \left( {\left( {P_{{{\text{b}}i}}^{\left( n \right)} + P_{{{\text{si}}}}^{\left( n \right)} } \right) + H_{{\text{d}}}^{\left( n \right)} \left( {P_{{{\text{b}}i}}^{{\left( {n - 1} \right)}} \varepsilon_{21i}^{\left( n \right)} + P_{{{\text{si}}}}^{{\left( {n - 1} \right)}} \varepsilon_{31i}^{\left( n \right)} } \right)} \right) + H^{\left( n \right)} P_{0i} = Z^{\left( n \right)} P_{{{\text{a}}i}}^{\left( n \right)}$$

where $Z^{\left( n \right)}$ is the thickness of the *n*th disturbance layer in the slurry ($Z^{\left( n \right)} = \overline{{V_{{\text{b}}} }} dt$), $H^{\left( n \right)} = H_{{\text{s}}}^{\left( n \right)} + H_{{\text{d}}}^{\left( n \right)}$ is the disturbance depth of the *n*th step, $H_{{\text{s}}}^{\left( n \right)}$ is the scouring thickness and $H_{s}^{\left( n \right)} = Z^{\left( n \right)} \left( {\mathop \sum \limits_{i = 1}^{\max } P_{si}^{{\left( {n - 1} \right)}} \varepsilon_{31i}^{\left( n \right)} + \mathop \sum \limits_{i = 1}^{\max } P_{bi}^{{\left( {n - 1} \right)}} \varepsilon_{21i}^{\left( n \right)} } \right)$, $H_{{\text{d}}}^{\left( n \right)}$ is the thickness of the upper deposition and $H_{d}^{\left( n \right)} = Z^{\left( n \right)} \left( {\mathop \sum \limits_{i = 1}^{\max } P_{ai}^{{\left( {n - 1} \right)}} \varepsilon_{13i}^{\left( n \right)} + \mathop \sum \limits_{i = 1}^{\max } P_{ai}^{{\left( {n - 1} \right)}} \varepsilon_{12i}^{\left( n \right)} } \right)$, $\varepsilon_{21i}^{\left( n \right)}$ and $\varepsilon_{31i}^{\left( n \right)}$ are the state transition probability, $P_{{{\text{a}}i}}^{\left( n \right)}$, $P_{{{\text{b}}i}}^{\left( n \right)}$, and $P_{{{\text{si}}}}^{\left( n \right)}$ are the gradations of the sediment load, the bedload, and the suspended load in the *n*th step, respectively, $\varepsilon$ is the weight percentage of sediment particles below the size of d_i_, $P_{0i}$ is the gradation of the initial slurry and $P_{{{\text{a}}i}}^{\left( 0 \right)} = P_{0i}$. Specifically, the second term on the left side of the equation is the scouring term, the third term is the sedimentation term, and the fourth term is the resanding term of the original slurry in the lower layer. The sand particle gradation of the *n*th step $P_{{{\text{a}}i}}^{\left( n \right)}$ can be expressed as follows^23^:

23 $$P_{ai}^{\left( n \right)} = P_{ai}^{{\left( {n - 1} \right)}} - \left( {H_{{\text{s}}}^{\left( n \right)} S_{{\text{s}}}^{\left( n \right)} - H_{{\text{d}}}^{\left( n \right)} S_{{\text{d}}}^{\left( n \right)} - H^{\left( n \right)} P_{0i} } \right)/Z^{\left( n \right)}$$

where the residual amount of fine particles in the *n*th step is $S_{{\text{s}}}^{\left( n \right)} = P_{{{\text{bi}}}}^{\left( n \right)} + P_{{{\text{si}}}}^{\left( n \right)}$, and the residual amount of fine particles in the *n*th step is ${ }S_{{\text{d}}}^{\left( n \right)} = P_{{{\text{bi}}}}^{{\left( {n - 1} \right)}} \varepsilon_{21i}^{\left( n \right)} + P_{{{\text{si}}}}^{{\left( {n - 1} \right)}} \varepsilon_{31i}^{\left( n \right)}$.

### Transition probability of slurry transport pattern

According to their transport state, particles under physical disturbance can be divided into separable sediment (state 1), bedload (state 2), and suspended load (state 3) that are convertible among them. Therefore, a three-state transition probability matrix of clay particles in sandy silty based on the concealment degree can be derived from the modification by Li et al.^23^ on the Markov nonhomogeneous discrete model proposed by Tsai and Wu^24^:

24 $$\varepsilon_{i}^{\left( n \right)} = \left[ {\begin{array}{*{20}c} {1 - P_{{{\text{Ti}}}}^{\left( n \right)} } & {P_{{{\text{Ti}}}}^{\left( n \right)} - P_{{{\text{Si}}i}}^{\left( n \right)} } & {P_{{{\text{Si}}i}}^{\left( n \right)} } \\ {1 - P_{2i}^{\left( n \right)} } & {P_{{{\text{Ri}}}}^{\left( n \right)} P_{2i}^{\left( n \right)} } & {P_{2i}^{\left( n \right)} \left( {1 - P_{{{\text{Ri}}}}^{\left( n \right)} } \right)} \\ {1 - P_{3i}^{\left( n \right)} } & {P_{3i}^{\left( n \right)} \left( {1 - P_{{{\text{si}}}}^{\left( n \right)} } \right)} & {P_{{{\text{Si}}}}^{\left( n \right)} P_{3i}^{\left( n \right)} } \\ \end{array} } \right]$$

where $\varepsilon_{i}^{\left( n \right)}$ is the probability matrix of the three-state transition of clay particles, $\varepsilon_{xy}^{\left( n \right)}$ represents the probability of particles transitioning from state X to state Y, $P_{{{\text{Ti}}}}^{\left( n \right)}$ is the total probability and $P_{{{\text{Ti}}}}^{\left( n \right)}$ = $P_{{{\text{Ri}}}}^{\left( n \right)}$ + $P_{{{\text{si}}}}^{\left( n \right)}$, $P_{{{\text{si}}}}^{\left( n \right)}$ is the motion initiation probability, $P_{{{\text{Ri}}}}^{\left( n \right)}$ is the transition probability of fine particles; and 1 − $P_{2i}^{\left( n \right)}$ and 1 − $P_{3i}^{\left( n \right)}$ are the rolling probability and suspension probability, respectively.

A clay particle transport pattern model in slurry under artificial disturbance is derived from the exposure/concealment effect-based sediment adjustment calculation model proposed by Li et al.^23^ and Eqs. (20) to (23):

25 $$P_{{{\text{a}}i}}^{\left( n \right)} = \left\{ {\begin{array}{*{20}l} {P_{{{\text{ai}}}}^{{\left( {n - 1} \right)}} - \alpha_{1} S_{{\text{s}}}^{\left( n \right)} + \alpha_{3} S_{{\text{d}}}^{\left( n \right)} + \left( {\alpha_{1} + \alpha_{3} } \right)P_{0i} } \hfill & { \left( {\Theta_{{\text{c}}} \Theta } \right)} \hfill \\ {P_{{{\text{ai}}}}^{{\left( {n - 1} \right)}} - \alpha_{2} S_{{\text{s}}}^{\left( n \right)} + \alpha_{3} S_{{\text{d}}}^{\left( n \right)} + \left( {\alpha_{2} + \alpha_{3} } \right)P_{0i} } \hfill & {(\Theta > \Theta_{{\text{c}}} )} \hfill \\ \end{array} } \right.$$

where the adjustment equation coefficients are $\alpha_{1} = V_{be}^{\left( n \right)} /V_{{\text{v}}}^{\left( n \right)} ,\alpha_{2} = H_{{\text{s}}}^{\left( n \right)} /{\Delta }_{{\text{z}}}^{^{\prime}\left( n \right)} ,\alpha_{3} = H_{{\text{d}}}^{\left( n \right)} /Z^{\left( n \right)}$.

## Discussion

In this paper, the indoor model test grading results of sandy silt compiled by Jia et al.^1^ are adopted, as shown in Table 1. The experimental research focused on two dredger filling modes. The first mode is one-time dredger filling to 50 cm and is disturbances at the depths of 5 cm, 15 cm, and 25 cm after 12 h, disturbances at the depths of 15 cm and 25 cm after 24 h, and disturbances at a depth of 25 cm after 36 h. The second mode is three-time dredger filling to 23 cm, 36 cm, and 50 cm at 0 h, 24 h, and 36 h, respectively, and disturbances at the depths of 5 cm and 15 cm at 0 h, disturbances at the depths of 15 cm and 25 cm after 24 h, and disturbances at the depths of 25 cm after 36 h. As the overlying loose soil layer was thick in the first mode, the disturbing force was 0.5 kPa, and the disturbing velocity $\overline{{V_{b} }} = 0.06 \;{\text{m/s}}$. In contrast, the overlying layers in the second mode were always 5 cm and 10 cm, the disturbing force was 0.2 kPa, and the disturbing velocity $\overline{{V_{b} }} = 0.08\;{\text{m/s}}$. The gradation is as follows.

**Table 1:** The gradation rate in the experiment^1^.

Water content (%)	Percentages of each gradation (%)					d50 (mm)
	< 0.05 mm	0.005–0.075 mm	0.075–0.125 mm	0.125–0.25 mm	> 0.25 mm	
60	15.6	74.81	5.97	3.41	0.21	0.038

Based on the grading results of indoor model tests on sandy silt, this study compares the three-state transition equation of uniform sand proposed by Li et al.^15^, Einstein’s formula^19^, and the bedload transport equation by Zhou^14^. The comparison results show that the equation of Li et al. did not consider the disturbance force reduction caused by the concealment between single particles, while Einstein's formula did not explain the threshold of particle motion initiation. Although the concealment effect is considered in Zhou’s equation, the sediment transport rate probability was only based on the motion initiation probability, and the particle transport state changes with the continued disturbance were neglected.

**Figure 6:**
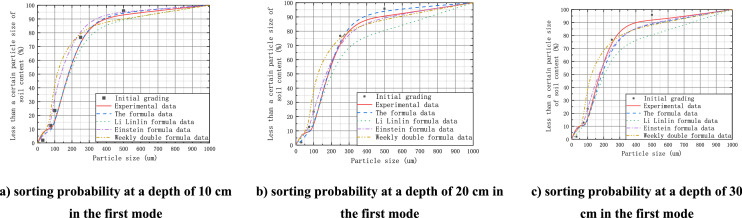
Comparison between calculated results and experimental data in the first pattern.

As shown in Fig. 6, thicknesses of the overlaying layers $H^{\left( n \right)}$ in the first mode were 10 cm, 20 cm, and 30 cm. Only the equation proposed in this paper and the transformed equation of Li et al. can facilitate analysis according to the relationship between affected layers of different thicknesses, while Einstein's formula and Zhou's bedload transport equation can only achieve comparison according to the calculation results of surface scouring. Analysis of the indoor model test results under artificial disturbance shows that the sediment clay content in the particle gradation after artificial disturbance is effectively reduced to 11.7%, and the clay particle removal effect is the best with the disturbance force at 20 cm, where the clay content is reduced to 10.4%. Judging from data discreteness, the calculated results of each equation are in a relatively consistent trend. However, the results of Einstein's formula and Zhou's equation show relatively large differences with the increase of depth. In addition, the results calculated with the equation of Li et al. tend to be relatively small with the increase of depth. Although the results are similar to those calculated in this paper, only the effect of surface disturbance is considered, the depth of the disturbance force is not, and the discreteness is relatively larger with particle size below 100 µm.

**Figure 7:**
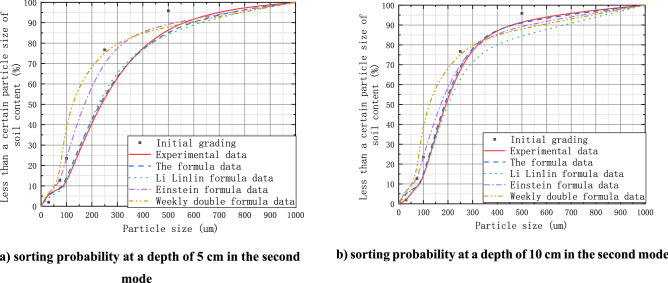
Comparison of calculated and experimental data in the second mode.

Comparative analysis of the sandy silt particle size characteristics after disturbance in the second mode (as shown in Fig. 7) indicates that the particle gradation changes caused by the disturbance force $P_{3i}^{\left( n \right)}$ ranges from 3.6 to 4.2%. The clay particle gradation difference before and after disturbance calculated with the equation in this paper is between 2.5 and 3.8%. However, the results calculated with Einstein's formula differed considerably from the measured data. Under the same disturbance intensity, the clay particle gradation has the largest variation. Although the goodness of fitting of the equation proposed in this paper and that of Li et al. is both high, the latter only considers the effects of unidirectional scouring. After multiple steps, the goodness of fitting is no longer high. In terms of the gradation difference of sand particles with large sizes during sorting, the gradation differences calculated with the equation of Li et al. differ considerably from the experimental data. In contrast, the calculated results in this paper have high goodness of fitting with the experimental data. Although the calculated results with Zhou's equation are more reasonable under disturbance intensities of ${{ \Theta }}$ (0.0001, 320) or ${\Theta }$ (0.04, 8.33), the calculated results for particles below 100 µm in size have relatively large differences with experimental data. The equation in this paper can effectively solve the problem of small particle motion initiation velocity due to the unidirectional concealment degree in the river scouring equation. With the Θc dimension to quantify the scouring effect of flow intensity on the particles, the calculated results are in good agreement with the flume experiment results.

## Conclusion

Based on the experimental results and theoretical derivation of the particle sorting pattern under artificial disturbance in sandy silt, the following conclusions were obtained.

1. The concept of bidirectional concealment degree was proposed according to the characteristics of silt formed at estuaries. The mechanical model of silt under disturbance was analyzed, and the mechanical model of silt particles under disturbance sorting state was constructed based on the relationship between the bidirectional concealment degree ${\Delta }_{x}^{^{\prime}}$ and ${\Delta }_{z}^{^{\prime}}$.
2. Shields parameter ${\Theta }$_c_ was adopted to calculate the motion initiation probability $\varepsilon_{{d_{i} }}$ of clay particles, the relationship between velocity ${ }\overline{{V_{{\text{b}}} }}$ and motion initiation probability ${ }\varepsilon$ under different water flow conditions was summarized, and Shields parameter ${{ \Theta }}$_c_ was introduced to calculate the motion initiation probability of clay particles. The depth of the disturbance layer and water flow conditions were classified by using the concept of ${\Theta }_{{{\text{cf}}}}$. Specifically, particle transport states were divided into the sediment, bedload, and suspended load. In addition, the Markov nonhomogeneous discrete model and the calculation model of sediment exposure/concealment adjustment proposed by Li et al. were effectively utilized to establish the particle transport pattern model in slurry under artificial disturbance.
3. Verification with the existing slurry particle sorting pattern under disturbance showed that although the riverbed sediment scouring gradation analysis equations (the classical Einstein’s formula, Zhou’s bedload analysis equation, and the three-state transition model equation of uniform sand particles by Li et al.) could effectively explain the bedload loss caused by single-side scouring from different aspects, single-side scouring had relatively limited effects on the flow conditions of particle motion initiation, suspension, and sedimentation and the particle transport sorting probability during clay particle removal from flow-plastic sandy silt by physical disturbance. The proposed equation considered not only the disturbance intensity, disturbance direction, and the particle concealment degree but also the depth of disturbance in the sedimentary slurry, thus effectively analyzing the grading calculation of sand silt particles after disturbance.
